# Exposure assessment of dietary cadmium: findings from shanghainese over 40 years, China

**DOI:** 10.1186/1471-2458-13-590

**Published:** 2013-06-18

**Authors:** Ping He, Yinhao Lu, Yihuai Liang, Bo Chen, Min Wu, Shuguang Li, Gengsheng He, Taiyi Jin

**Affiliations:** 1 Department of Nutrition and Food Hygiene, School of Public Health, Key Laboratory of Public Health Safety. Ministry of Education, Fudan University, 138 Yi Xue Yuan Road, Shanghai 200032, China; 2 Department of Occupational Health and Toxicology, School of Public Health, Key Laboratory of Public Health Safety, Ministry of Education, Fudan University, 138 Yi Xue Yuan Road, Shanghai 200032, China

**Keywords:** Cadmium, Non-occupational exposure, Food intake, Exposure assessment

## Abstract

**Background:**

Environmental exposure to cadmium causes renal dysfunction and bone damage. Cadmium contamination in food is regarded as the main environmental source of non-occupational exposure. The aim of this study was to assess the contribution of dietary cadmium exposure in environmental cadmium exposure and its health risk among adults in Shanghai, China.

**Methods:**

A cross-sectional survey about food consumption was conducted in 2008 among 207 citizens aged over 40 years in Shanghai, China. The food frequency questionnaire was combined with food, tobacco and water cadmium exposure to estimate the daily environmental cadmium exposure in both point and probabilistic estimations. Urine and blood samples of the participants were analyzed for internal exposure to total cadmium. Correlation analysis was conducted between the internal cadmium exposure and environmental cadmium exposure.

**Results:**

According to the point estimation, average daily environmental cadmium exposure of the participants was 16.7 μg/day and approached 33.8% of the provisional tolerable daily intake (PTDI). Dietary and tobacco cadmium exposure approached 25.8% and 7.9% of the PTDI, respectively. Males had higher levels of dietary cadmium exposure than females (p?=?0.002). The probabilistic model showed that 93.4% of the population did not have any health risks from dietary cadmium exposure. By sensitivity analysis, tobacco consumption, tobacco cadmium level, cadmium in vegetables and cadmium in rice accounted for 27.5%, 24.9%, 20.2% and 14.6% of the total cadmium exposure, respectively. The mean values of urinary and blood cadmium among the study population were 0.5 μg/L and 1.9 μg/L, respectively. Positive correlations were observed between environmental cadmium exposure and blood cadmium (R?=?0.52, P<0.01), tobacco cadmium intake and blood cadmium excluding non-smokers (R?=?0.26, P?=?0.049<0.05), and urine cadmium and age (R?=?0.15, P?=?0.037).

**Conclusions:**

It has been suggested that there is no increased health risk among adult residents in Shanghai, China because of recent total cadmium exposure. Vegetables and rice were the main sources of dietary cadmium intake. Tobacco cadmium exposure, which accounted for approximately 25% of the total dietary cadmium exposure, was another important source of non-occupational cadmium exposure.

## Background

Cadmium is one of the trace metals widely used in different types of industries. However, cadmium can be a source of soil and water pollution after extraction and discharge. Cadmium contamination is a major environmental health problem, and is potentially harmful to the broader population through the food chain [1]. The half-life of cadmium is 10 to 30 years in humans. Cadmium slowly accumulates in organs, such as the kidneys and bone, and causes chronic damage, such as renal dysfunction and fractures [2]. Long-term exposure to low-dose cadmium has been associated with increased oxidative stress and tubular impairment [3,4]. Additionally, there is a dose–response relationship between urinary cadmium levels and some chronic diseases, such as diabetes and high blood pressure [5,6]. In 1993, IARC classified cadmium and its compounds as human carcinogens (primarily lung cancer) [7]. Recent studies have shown that dietary cadmium exposure is associated with the development of postmenopausal breast cancer in women [8].

To prevent health risk from cadmium contamination, a provisional tolerable weekly intake (PTWI) for cadmium of 7 μg/kg body weight was established by the Joint FAO/WHO Expert Committee on Food Additives in 2004 [9]. In 2010, the 73rd JECFA reevaluated cadmium intake levels based on findings from a number of recent epidemiological studies and established a provisional tolerable monthly intake (PTMI) of 25 μg/kg body weight based on the long half-life of cadmium [10].

Cadmium contamination is severe and prevalent in some areas of China. As reported, food is the main source of cadmium exposure for the non-occupationally exposed population (WHO 1992). In recent years, it has been reported that cadmium levels in some food in Shanghai have exceeded the National Maximum Level as follows: 27.2% in aquatic products, 2.7% in animal innards and 8.2% in grains [11]. Therefore, it was necessary to evaluate dietary cadmium exposure and its health risk for future policymaking on cadmium contamination prevention.

This study aimed to assess dietary cadmium exposure and other environmental cadmium exposure compared with the PTWI standard among adult residents (over 40 years of age) in Shanghai, China, and to determine the major contributors of total cadmium exposure. Food, tobacco and water cadmium levels were combined to evaluate the external cadmium exposure, and internal exposure levels were assessed. Therefore, this study may provide scientific evidence for cadmium monitoring, risk management and policymaking.

## Methods

A cross-sectional survey concerning food consumption was conducted among Shanghai residents. The study was approved by the institutional review board of the School of Public Health, Fudan University. Informed consent from participants was obtained prior to enrollment in the study.

### Food consumption survey and internal exposure measurement

In July 2008, a survey of residents was conducted to collect food consumption data and measure internal cadmium exposure. The participants were recruited from those who routinely attended health checks at the Songnan Town Community Health Center (CHC), Baoshan District, Shanghai, China. Convenience sampling was used for recruitment, and 267 study participants were selected. Participants over 40 years old were chosen because of the accumulation effect of cadmium in middle-aged and senior humans. Participants who had been diagnosed with serious kidney, bone and genetic diseases were excluded. Demographic data were collected by questionnaire. Information on food intake was collected using a reliable and valid food frequency questionnaire (FFQ) that was from the National Nutrition Survey [12]. Thirty-nine common food items were included in the questionnaire. Participants were asked about their intake frequency of each food per day (week/month/year) and the amounts ingested each time over the past year. The food was categorized as follows: rice, wheat flour, cereals (millet, sorghum and maize), tubers (sweet potato, yam, taro and potato), pork, bacon, salted pork, other meat product, pork offal (lung, stomach, kidney, intestine, liver and other offal), beef and mutton, poultry meat, aquatic products, milk, milk powder, cheese, yogurt, egg, tofu, soybean products, soya-bean milk, dried beans, fresh vegetable, salted vegetables, vegetables pickled in soy sauce, kimchi, pastries, fresh fruit, nuts, liquor (below 38% alcohol), liquor (above 38% alcohol), beer, fruit wine, fruit juice beverages, and other beverages. Moreover, daily cigarette consumption was investigated to differentiate smokers from non-smokers and calculate tobacco cadmium exposure.

Venous whole blood samples were collected in heparin tubes; urine samples were collected in acid-washed containers to avoid inside contamination and acidified with concentrated nitric acid following guidelines of sample collection. Both sample types were stored at -20°C [13]. Urinary cadmium (UCd) and blood cadmium (BCd) were tested using national standard methods by WS/T 32–1996 Graphite furnace atomic absorption spectrometry (GFAAS, Shimadzu AA-670, Kyoto, Japan), which has been described by Jin and Chen [14,15]. Four water samples were collected from local waterworks, and water cadmium was detected by the same method using GFAAS. The limit of detection (LOD) of both urine and blood cadmium was 0.05 μg/L [15].

### Estimation of cadmium Intake and Internal Exposure

Both point and probabilistic estimations were used to calculate cadmium intake and the contribution of various food items to the exposure in this study. The method followed FAO and WHO guidelines [9].

The point estimation method was applied to calculate individual exposure. The Statistical Package for the Social Sciences (SPSS, version 12) was used to analyze consumption and cadmium data. The average body weight of was approximately 60 kg and displayed a normal distribution, and a PTMI of 25 μg/kg body weight (JECFA, FAO/WHO 2010) was converted into a PTDI of 49.5 μg/day as the study criteria.

Dietary exposure formulas are listed as follows:

Dietary cadmium exposure?=?Σ (cadmium in food?×?average food consumption)

Mean dietary cadmium exposure?=?mean cadmium concentration of food?×?mean dietary intake

Median dietary cadmium exposure?=?mean cadmium concentration of food?×?median dietary intake

Upper 90th percentile dietary cadmium exposure (P90)?=?mean cadmium concentration of food?×?dietary intake P90

Extreme 90th percentile dietary cadmium exposure (extreme P90)?=?cadmium concentration of food P90?×?dietary intake P90

Food contribution rate of cadmium exposure?=?(cadmium exposure of the food/total dietary cadmium exposure)?×?100%

In the FFQ, which was developed by staff at the Shanghai Municipal Center for Disease Control and Prevention (SCDC), 1,680 food items were included using clustering random sampling from various districts of Shanghai during 2002 and 2007 [16]. GFAAS was used to determinate the food cadmium levels, which was described in a Chinese national cadmium exposure survey in 2000 [17,18].

Tobacco and water consumption were also collected for the total cadmium exposure calculation. Tobacco cadmium through inhalation was one of the most important non-occupational resources, and based on study by Qian et al., the cadmium concentration was assumed to be 1.5 mg Cd/kg [19]. The smoking cadmium, water cadmium exposure and total cadmium exposure in the population was calculated as follows:

Smoking cadmium exposure?=?mean tobacco cadmium in each cigarette?×?mean smoking cigarettes?×?mean smoking years

Water cadmium exposure?=?mean water cadmium level?×?mean drinking water

Total cadmium exposure?=?dietary cadmium exposure?+?smoking cadmium exposure?+?water cadmium exposure

A probabilistic estimation method was applied to evaluate population exposure. Crystal ball software 11 was used to perform a Monte Carlo simulation, which is widely used by the US Environmental Protection Agency (EPA). On the basis of the guiding principles for Monte Carlo analysis [20], the population cadmium intake was simulated according to the above specified total cadmium exposure formula. Monte Carlo simulation is a computational method that relies on repeated random sampling to obtain numerical results, such as probabilistic distribution. Population-based exposure and risk distributions were estimated using simulated values (n?=?100,000).

Correlation analysis between environmental cadmium exposure and total internal cadmium was calculated by SPSS 12.0. External and internal cadmium differences by gender and smoking status were compared by Mann–Whitney U Test because the data was non-normally distributed. P value < 0.05 was considered a significant difference between the compared groups.

## Results

### Dietary exposure and internal exposure

The participant characteristics are shown in Table 1. Two hundred and seven participants remained in the study excluding those who had incomplete information, such as a lack of blood or urine samples. For smokers, 57 were men, and 4 were women.

**Table 1:** **Characteristics of research participants in Shanghai**, **China 2008**

	**Number**	**Percentage (%)**
**Age**		
<60 years	130	62.8
≥60 years	77	37.2
**Gender**		
Male	86	41.5
Female	121	58.5
**Marriage**	190	91.8
**Education > 9years**	160	77.3
**Smokers**		
smoking	61	29.5
never	146	70.5
**Diabetic**	17	8.2

Cadmium levels of food items and participants’ cadmium intake are listed in Table 2, and the calculated dietary exposure is presented in Table 3. The mean dietary cadmium exposure was 12.8?±?4.2 μg/day. Vegetables, rice and seafood were the three main sources of dietary cadmium exposure and accounted for 86.3% of the total exposure (40.2% from vegetables, 37.6% from rice and 8.5% from seafood).

**Table 2:** **Cadmium level of local food** (**mg**/**kg**) **and daily intake of local food** (**g**/**day**)

	**Cadmium detection (mg/kg)**^1*^				**2000 Survey**^2*^	**Daily intake (g/day)**			
	**Mean**	**Std**	**Median**	**P90**	**Mean**	**Mean**	**Std**	**Median**	**P90**
Rice	0.023	0.031	0.009	0.09	0.008	208.5	102.6	200.0	300.0
Wheat	0.014	0.015	0.011	0.023		33.3	48.0	14.3	100.0
Coarse cereal	0.006	0.007	0.006	0.02		6.8	10.7	3.3	14.3
Tuber	0.002	0.001	0.001		0.015	7.4	11.4	1.7	24.3
Pork	0.018	0.035	0.002	0.1	0.572	27.6	39.9	14.3	55.0
Innard	0.278	0.607	0.001	0.006		0.5	1.7	0.0	1.7
Fatstock except pork	0.003	0.003	0.001	0.006		4.7	7.2	1.7	14.3
Poultry	0.002	0.001	0.001	0.003		11.5	15.6	7.1	28.6
Aquatic product	0.043	0.225	0.007	0.091	0.024	25.3	31.3	14.3	50.0
Egg	0.005	0.006	0.003	0.012	0.004	28.4	30.8	21.4	50.0
Milk	0.001	0.001	0.001	0.003	0.002	99.3	122.9	35.7	262.9
Dry bean	0.019	0.03	0.007	0.1	0.023	12.5	47.5	1.7	14.3
Fresh vegetable	0.025	0.05	0.005	0.17	0.026	205.3	106.7	200.0	300.0
Fruit	0.001	0.002	0.001	0.003	0.002	63.9	58.3	50.0	150.0

1* Cadmium detection data were from a Dietary Cadmium Exposure Assessment in Shanghai Adult Residents 2010 [16].

2* Mean data were cadmium levels in Shanghai in 2000 from a Chinese National Food Survey [18].

**Table 3:** **Sources of environmental cadmium exposure among study participants in Shanghai**, **China 2008**

**Types**	**Mean**		**Median**		**Dietary intake P90**		**Extreme P90**	
	**Daily exposure (μg/day)**	**Contribution rate (%)**	**Daily exposure (μg/day)**	**Contribution rate (%)**	**Daily exposure (μg/day)**	**Contribution rate (%)**	**Daily exposure (μg/day)**	**Contribution rate (%)**
Rice	4.80	37.56	1.80	54.91	6.90	33.54	27.00	28.67
Flour	0.47	3.65	0.16	4.79	1.40	6.81	2.30	2.44
Coarse cereal	0.04	0.32	0.02	0.61	0.09	0.42	0.29	0.30
Tuber	0.01	0.12	0.00	0.05	0.05	0.24	0.10	0.10
Pork	0.50	3.89	0.03	0.87	0.99	4.81	5.50	5.84
Innard	0.15	1.19	0.00	0.00	0.46	2.26	0.01	0.01
Fatstock*	0.01	0.11	0.00	0.05	0.04	0.21	0.09	0.09
Poultry	0.02	0.18	0.01	0.22	0.06	0.28	0.09	0.09
Seafood	1.09	8.51	0.10	3.05	2.15	10.45	4.55	4.83
Egg	0.14	1.11	0.06	1.96	0.25	1.22	0.60	0.64
Diary product	0.10	0.78	0.04	1.09	0.26	1.28	0.79	0.84
Dry bean	0.24	1.87	0.01	0.36	0.27	1.32	1.43	1.52
Vegetable	5.13	40.21	1.00	30.51	7.50	36.46	51.00	54.15
Fruit	0.06	0.50	0.05	1.53	0.15	0.73	0.45	0.48
Dietary Exposure	12.77	100.00	3.28	100.00	20.57	100.00	94.18	100.00
Water Exposure	0.03		0.03		0.05		0.05	
Smoking Exposure	3.93		0.00		16.15		16.39	
Daily Exposure	16.73		3.31		36.78		110.63	

*Fatstock represents fatstock except pork.

The dietary cadmium exposure was higher in men (mean?=?14.0 μg/day) than in women (mean?=?11.9 μg/day) with a significant difference (p?=?0.002). For men, rice, vegetables and seafood were the three main sources of dietary cadmium exposure and accounted for 38.6%, 36.4% and 10.2% of the total exposure, respectively. For women, the ranking was slightly different; vegetables, rice and seafood were the top three exposure sources (44.1%, 36.5% and 6.7%, respectively).

**Figure 1:**
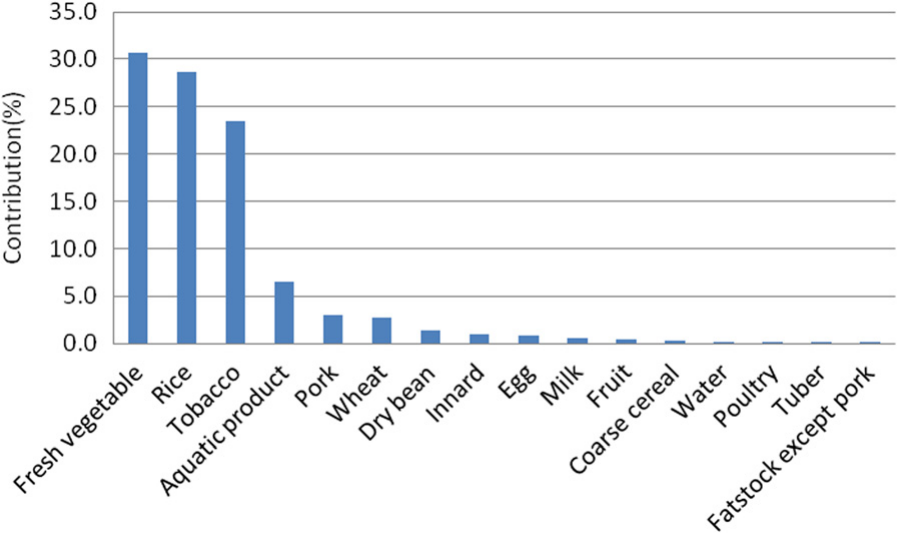
Contribution of cadmium factors to daily cadmium exposure (%).

Tobacco cadmium exposure was 3.9 μg/day (range from 0–73.1 μg/day). The median and P95 were 0 and 18.8 μg/day, respectively. For smokers, the mean tobacco cadmium exposure was 13.8?±?12.3 μg/day which almost equaled to the dietary cadmium exposure (14.0?±?4.8 μg/day). The cadmium levels of water samples were lower than the LOD (0.05 μg/L). Therefore, we assumed that the local water cadmium level was half of the LOD (0.025 μg/L), and participants drank 1200 mL water daily. Accordingly, the mean water cadmium exposure was 0.03 μg/day and accounted for 0.2% of the total environmental cadmium exposure. As shown in Figure 1, the three main exposure factors were vegetables (30.6%), rice (28.5%) and seafood (23.5%). These food items accounted for 82.6% of the total environmental cadmium exposure.

Mean urine cadmium (UCd) and blood cadmium (BCd) of the study participants were 0.52 μg/L and 1.88 μg/L, respectively. As shown in Table 4, there was a statistically significant difference in blood cadmium between men and women (p?<?0.001), but no such difference in urine cadmium.

**Table 4:** **UCd and BCd** (**μg**/**L**) **of participants by gender and smoking status in Shanghai**, **China 2008**

BCd*	**n**	**Mean**	**Std. Deviation**	**Median**	**P95**
Male	86	0.75	0.81	0.43	2.49
Female	121	0.36	0.34	0.28	1.04
Total	207	0.52	0.61	0.31	1.77
**UCd**					
Male	87	1.80	1.52	1.38	5.50
Female	121	1.94	1.43	1.64	4.95
Total	207	1.88	1.47	1.54	5.12
BCd*					
Smoke	61	1.04	0.86	0.91	3.32
None smoke	146	0.30	0.25	0.27	0.65
**UCd**					
Smoke	61	1.94	1.55	1.51	5.60
None smoke	146	1.86	1.44	1.57	5.02

* Mann–Whitney U Test, P<0.01.

Because smoking status was one of the exposure factors, participants were classified into the smoking group and the non-smoking group, and cadmium was detected both in urine and blood samples (Table 4). There were significant differences in blood cadmium concentrations between the two groups (P?<?0.001) whereas no difference in urine cadmium levels was observed.

**Figure 2:**
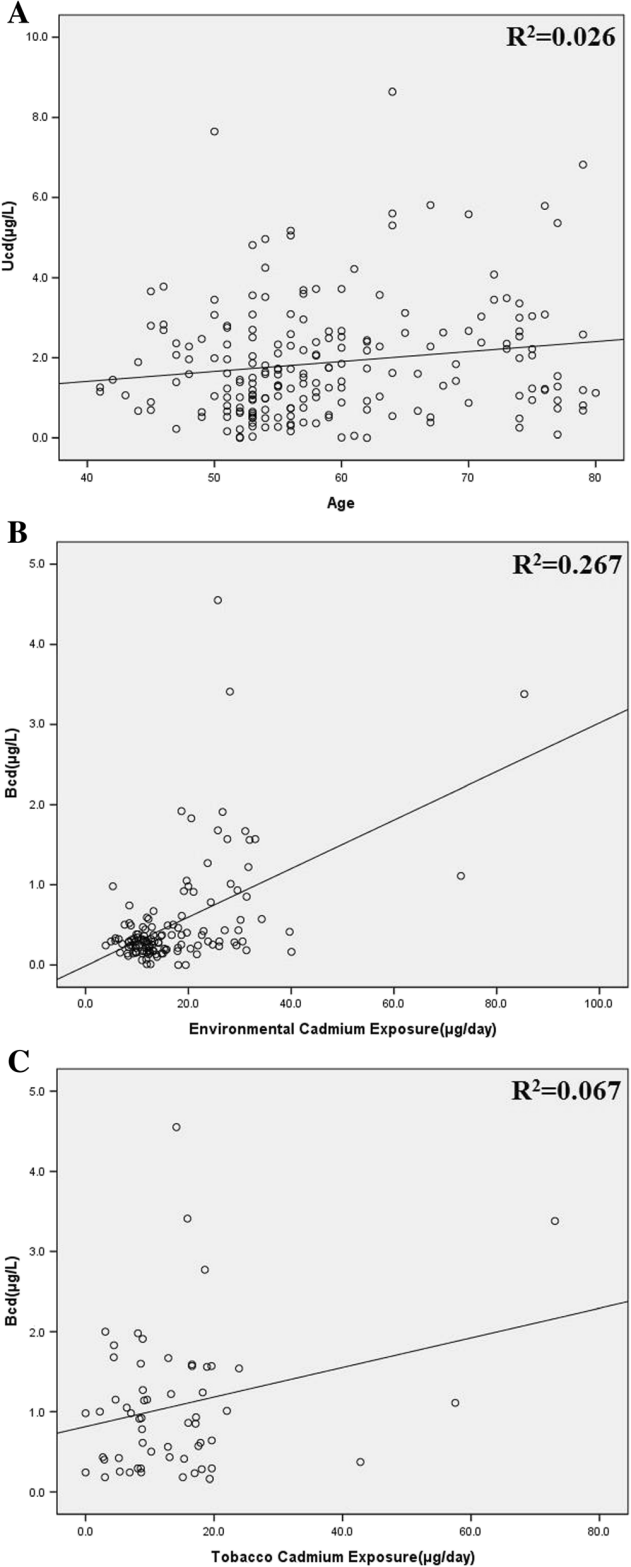
**Simple scatter plots of UCd-Age, BCd-environmental cadmium exposure and BCd-tobacco cadmium intake. (A)**: UCd-Age **(B)**: BCd-Environmental Cadmium Exposure **(C)**: BCd-Tobacco Cadmium Intake.

A positive correlation between urine cadmium levels and age was observed (R?=?0.15, P?=?0.037). There was also a positive correlation between environmental cadmium exposure and blood cadmium levels (R?=?0.52, P<0.01). Excluding the non-smokers, tobacco cadmium intake was positively correlated with blood cadmium levels (R?=?0.26, P?=?0.049). Simple scatter plots are shown in Figure 2.

### Assessment of Cadmium Exposure

#### *Point Estimation of Environmental Cadmium Exposure*

The mean and median total cadmium exposures among the adult study participants were 16.7 μg/day and 3.3 μg/day and accounted for 33.8% and 6.7% of the PTDI, respectively. The dietary intake extreme P90 was 110.6 μg/day and was 223.5% of the PTDI. The mean dietary and tobacco cadmium exposure approached 25.8% and 7.9% of the PTDI.

Among male participants, the mean and median total cadmium exposures were 17.8 μg/day and 3.5 μg/day, which were 36.0% and 7.1% of the PTDI, respectively. The dietary intake P90 was 42.23 μg/day, which was 85.3% of the PTDI. For female, the mean and median dietary cadmium exposures were 15.8 μg/day and 3.34 μg/day, which were 31.9% and 6.7% of the PTDI, respectively. The dietary intake P90 was 35.7 μg/day, which was 72.1% of PTDI.

#### *Probabilistic Estimation of the Population Environmental Cadmium Exposure*

**Figure 3:**
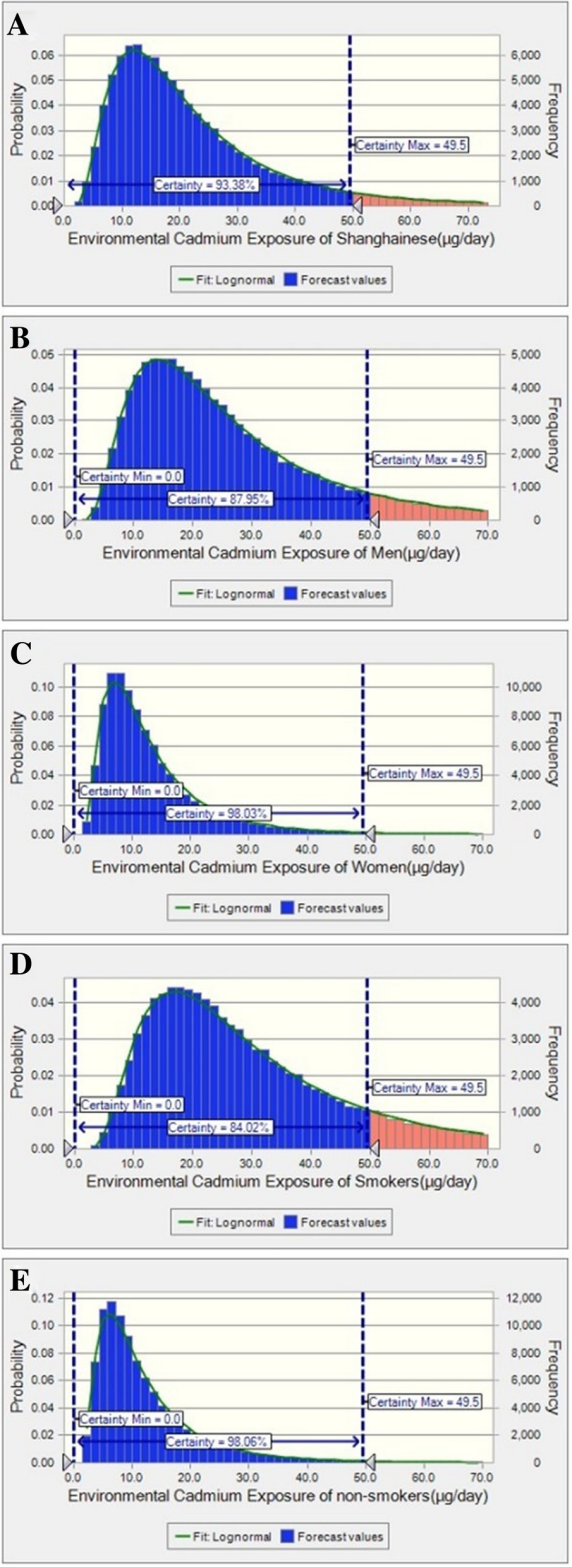
**Probabilistic risk model of dietary cadmium exposure for adults in Shanhai, China (μg/day). (A)**: Environmental Cadmium Exposure (ECd) of Inhabitants (μg/day). **(B)**: ECd of men **(C)**: ECd of women. **(D)**: ECd of smokers **(E)**: ECd of non-smokers.

The Monte Carlo method was used to calculate population exposure distributions by fitting the suitable distributions of the input variables in the dietary cadmium exposure equations. Frequency histograms from the simulation for cadmium are presented in Figure 3, and probabilistic distributions and statistics of daily cadmium exposure are shown in Table 5. Lognormal distribution was the best fitting distribution to this equation. The probability of population cadmium intake below the PTDI value was 93.4% and ranged from 0 to 49.5 μg/day. The mean cadmium intake was 23.1?±?18.0 μg/day by probabilistic analysis.

**Table 5:** **Probabilistic distributions and statistics of daily cadmium exposure** (**μg**/**day**)

**Model**	**Type of distribution**	**Mean**	**Median**	**STD**	**5th-95th percentile**	**Range**
Total	Lognormal	23.05	18.24	18.02	6.8-55.1	1.7-376.0
Men	Lognormal	28.49	22.2	23.05	7.7-69.0	1.5-457.8
Women	Lognormal	14.05	10.62	12.66	4.2-34.5	1.3-314.0
Smokers	Lognormal	32.74	25.78	25.83	9.6-78.3	2.8-556.1
Non-smokers	Lognormal	13.23	9.63	12.72	3.6-34.0	1.0-356.44

Sensitivity analysis was also conducted for the probabilistic risk model based on different types of food items. The food items with the highest sensitivity were tobacco cadmium level (27.5%), tobacco consumption (24.9%), vegetables (20.2%) and rice (14.6%).

The probability of environmental cadmium exposure below the PTDI value was 88.0% for males and 98.0% for females. Sensitivity analysis of the probabilistic risk model by gender found the following: for males, factors of highest sensitivity were cadmium in tobacco (37.2%), tobacco consumption (30.4%), cadmium in vegetables (12.3%) and cadmium in rice (11.2%), whereas for females, the factors were cadmium in vegetables (42.8%), cadmium in rice (30.3%), vegetable intake (7.8%) and rice intake (6.7%).

The probability of environmental cadmium exposure below the PTDI value was 84.0% for smokers and 98.1% for non-smokers. The factors of highest sensitivity for smokers were cadmium in tobacco (42.6%), tobacco consumption (28.9%) and cadmium in vegetables (10.2%), whereas for non-smokers, the factors were cadmium in vegetables (41.8%), cadmium in rice (33.1), rice intake (8.5%) and vegetable intake (7.3%). The non-smoking men and non-smoking women were compared, and the risks were 2.25% and 1.85% above the PTDI, respectively. Cadmium in vegetables (42.7%), cadmium in rice (36.0%), rice consumption (9.3%) and cadmium in seafood (6.5%) were sensitive for non-smoking men. Cadmium in vegetables (44.9%), cadmium in rice (31.3%), vegetable consumption (7.8%) and rice consumption (6.9%) were sensitive for non-smoking women.

## Discussion

This study provided a detailed assessment of environmental cadmium exposure, especially dietary exposure among middle-aged and senior residents in Shanghai, China. We found that the mean dietary cadmium exposure level of the population was far below the PTDI value, and a very low percentage of the population (6.6%) exceeded the PTDI. Moreover, 93.4% of the residents had no health risks, even if they had consumed all types of food with detectable cadmium levels throughout their lifetime. Men had higher dietary cadmium exposure levels than women, which could be explained by smoking and dietary behaviors, especially consuming food with high cadmium concentrations, such as vegetables and grains. Because the food supply in Shanghai does not meet local demand and much is imported from other provinces or other Asian countries, the food cadmium level did not simply reflect the local pollution, but that of a much wider area. These data could also represent pollution conditions in other non-polluted coastal areas in China other than Shanghai because of similar dietary patterns.

To better understand the food cadmium administration condition in China, the Chinese food cadmium maximum limits (MLs) are listed in Table 6. The Chinese MLs quoted national standard and international MLs were from WHO and European Food Safety Authority (ESFA) [21-25]. The rice MLs in China were lower than those of the WHO. Rice cadmium levels in our study were much less than the MLs in China (90th percentile?=?0.09 mg/kg). However, it is necessary to reevaluate whether the MLs of rice should be increased to 0.4 mg/kg because rice is the main food of Chinese, and many planting areas exhibit cadmium pollution in China. The meat MLs were higher than those of the WHO. In this study, the 90th percentile of cadmium levels in bovine animals was 0.1 mg/kg, which could be caused by polluted feed and contamination in processing. Higher fish MLs may be caused by water and feed pollution. Table 2 shows that food cadmium levels in this study were lower compared with levels in 2000 in Shanghai, which might indicate a reduction in cadmium contamination.

**Table 6:** **Comparison of cadmium maximum levels** (**MLs**) **in foodstuffs** (**mg**/**kg**)

	**China**	**ESFA**	**WHO**
Rice, soybean	0.2	0.2	0.4
Wheat, bran, germ	0.1	0.2	0.2
Other cereals	0.1	0.1	0.1
Peanut	0.5	0.1	0.1
Vegetables and fruit, excluding leaf	0.05	0.05	0.05
Vegetables, fresh herbs, fungi, stem			
Vegetables, root vegetables, and potatoes			
Stem vegetables, root vegetables and potatoes, excluding celeriac.	0.1	0.1	0.1
Leaf vegetables, fresh herbs, celeriac and common mushroom	0.2	0.2	0.2
Meat (excluding offal) of bovine animals, sheep, pig, and poultry	0.1	0.05	0.05
Liver of bovine animals	0.5	0.5	0.5
Kidney of bovine animals	1	1	1
Muscle meat of fish, excluding Bonito and bullet tuna	0.1	0.05	0.05-1
Crustaceans		0.5	0.05-1
Bivalve molluscs and Cephalopods		1	2
Egg	0.05		

The local total daily intake (TDI?=?12.73 μg/day) of cadmium was lower than the levels in other rice-dependent Asian countries. The cadmium TDI of Bangladeshis was estimated as 34.55 μg/day, which was 57.8% of the PTDI [26]. Average daily cadmium exposure was 14 μg in the South Korean population, representing approximately 27% of the TDI [27]. Dietary cadmium intake of the general Japanese population was approximately 25 to 30 μg/day [28]. Other units of exposure, such as kg per bodyweight, are used as the unit of cadmium exposure in Western countries. Therefore, for better comparability, these data were converted by assuming an adult average bodyweight of 60 kg. The TDI was 22.2 μg/day in Germany [29], 8.4 μg/day in Catalonia (Spain) [30], 8.6 μg/day in Sweden [31], 8.4 μg/day in Belgium [32] and 20.9 μg/day in France [33]. In these countries, the highest contribution to total cadmium intake corresponded to tubers, such as potatoes, cereals, and pulses, and aquatic products [29-35]. Although rice, which is the main food in Shanghai, was prone to cadmium accumulation, the data did not show a significant difference between Shanghai and Western countries in cadmium accumulation in other foods.

Compared with other cities in China, the local total daily intake (TDI?=?12.73 μg/day) of cadmium in Shanghai was lower than that in the Guangdong province (43.22 μg/day), where the soil cadmium background value was higher than other places [36] but slightly higher than Wuhan (10.8 μg/day), another non-polluted city in the middle of China [37]. The average TDI was 15.25 μg/day for adults in northern China and 27.6 μg/day in three southern areas (Shanghai, Jiangxi Province and Fujian Province) according to Chinese national dietary research in 2000 [17]. One reason for the decrease in food cadmium exposure might be because intake of certain foods, especially rice, has been decreasing in the past decades due to changing dietary patterns in China [38]. Our study found that vegetables, rice and aquatic products contributed most to dietary cadmium exposure, which was consistent with findings from Chinese national dietary research in 2000 [18]. The relative dietary contributions differed in other regions, indicating dietary pattern differences. Sensitivity analysis showed that cadmium in vegetables contributed most to dietary cadmium exposure, followed by rice and aquatic products. Other Asian studies have found that cadmium in rice and vegetables contributed most to dietary exposure [26,27]. Therefore, controlling the cadmium concentration in these particular food items could most likely reduce dietary cadmium exposure in Shanghai.

Of the total population older than 15 years, 35.8% were smokers according to the National Survey in 2002 [39]. The percentage of men and women smokers were 66.0% and 3.1%, respectively, which was confirmed by our study (66.2% for men and 3.3% for women). Tobacco cadmium through inhalation is another important source of non-occupational cadmium exposure, of which the concentration could be approximately 0.5 to 5 mg/kg [40]. According to this study, exposure through tobacco cadmium inhalation appeared to be the second greatest source of non-occupational cadmium exposure in the population. Our study demonstrated that tobacco cadmium inhalation accounted for 25% of the food cadmium intake and correlated with BCd. Smoking inhabitants were 14.0% more likely to exceed the PTDI compared with non-smokers. The study also confirmed that tobacco cadmium exposure (13.8 μg/day) was approximately equivalent to the total dietary cadmium exposure (14.0 μg/day). The same conclusion was found by Van Assche who constructed a model indicating that approximately 50% of the total cadmium intake was derived from cigarettes in smokers, whereas ingestion accounted for 95% of total cadmium intake in non-smokers [41]. Madeddu et al. concluded that smoking was the most important risk factor for blood cadmium concentration in the selected population [42]. National Health and Nutrition Examination Surveys (NHANES) from 1988 to 2008 indicated that declining smoking rates and changes in exposure to tobacco smoke might have played an important role in the decline of urine cadmium concentrations [43]. Additionally, a significant and higher difference in blood cadmium concentrations was observed in smokers compared with non-smokers. The study indicated that one effective way to decrease the risk of non-occupational cadmium exposure might be to reduce the smoking rate.

External exposure represented a cadmium exposure from environmental sources, whereas the total internal cadmium was estimated in body organs and the bloodstream. There is no unified diagnostic cadmium criterion for the general population in China. The diagnostic criterion of occupational cadmium poisoning (GBZ17-2002) is 5 μg/g creatinine (5 μg/L) for urinary cadmium, whereas the discriminant standard for health hazard caused by environmental cadmium pollution (BGT17221-1998) is 15 μg/g creatinine, which is the recommended upper limit for an environmental cadmium diagnosis in China [44]. Moreover, American Conference of Industrial Hygienists established a similar standard that the biological exposure index for cadmium is 5 μg/L in blood and 5 μg/g creatinine in urine for workers [45]. A health-based limit of 10 μg/L cadmium in blood and 10 μg/g creatinine in urine was established by the WHO [46]. Local participants did not appear to be adversely effected by recent environmental exposure according to urinary cadmium levels, which reflected the body burden over long-term exposure. Body cadmium levels (i.e., UCd) increased with age because of the accumulation of cadmium. A positive correlation between BCd and environmental cadmium exposure indicated recent exposure status.

Exposure assessment, which is an important step in risk assessment, describes the contaminant level in humans instead of extrapolating from animal models. One of the strengths of our study is that we conducted both point and probabilistic estimations to assess cadmium intake including from water and tobacco. Many other references assessed single food items or used point estimations only. For example, the national cadmium exposure survey 2000 in China selected 12 provinces to assess total dietary cadmium exposure, but performed only point estimates without cadmium detection in urine and blood. The advantage of using probabilistic estimation is that all of the available information about variability and inherent uncertainty are included in the assessment, whereas for point estimation most of the information about the variability and uncertainty is discarded, and only one point value is selected [47]. Distribution functions for exposure or risk estimates display a range of exposure or risk and the probability associated with each value. Second, the point estimation may calculate only the conservative or high-end estimated value, which may be far above realistic estimates when determining an upper bound of risk. However, the probabilistic estimation provides a reasonable value that is calculated by the distribution of exposure [48]. Third, sensitivity analysis can be used in the probabilistic estimation to assess the contribution of each risk factor. More data and accurate assumptions are required to build a probabilistic estimation model, which is a shortcoming. We applied Monte Carlo simulation for probabilistic assessment to analyze the cadmium dietary exposure distribution and compared the contributions of different food types with exposure. This model is a widely used computational method with risk parameters, and was first described in 1949 by Metropolis and Ulam [49]. It can be concluded that with appropriate data and simulation conducted with a sufficiently large number of “iterations”, the results will simulate actual conditions [9]. Furthermore, BCd and UCd were collected in the study to directly evaluate cadmium accumulation in the body and illustrate potential health outcomes.

This study had some limitations that should be considered in further investigations. Considering the precision and validity of the FFQ, we selected adult participants (age?>?40 years) but excluded children who were also susceptible to health hazards, such as early kidney toxicity and neurotoxic effects, from cadmium exposure [50]. Major food categories were considered in the food safety audit, but specific food items, which are rarely consumed, were not excluded. Therefore, the results might be slightly lower than the actual level for the PTDI. Cadmium exposure during food processing, which might have led to an overestimated exposure level, was not considered. Biomarkers that indicated early renal dysfunction were not measured. Other trace metals were not detected in this study and might have similar mechanisms of action as cadmium. The Monte Carlo simulation, which depends on the accuracy of parameters, may be inaccurate at the extreme upper and lower ends of the distribution [9]. A larger sample size might have allowed the use of a more suitable probabilistic distribution model.

This study assessed total cadmium exposure including food, water and tobacco in a non-occupational population. The dietary structure of the Chinese is complex, and chemical toxicant concentrations vary by food type. Therefore, the dietary survey conducted in this study helped identify items accurately and calculated the total amount of cadmium contaminant exposure. The risk level of inhabitants was determined by point and probabilistic estimations. Overall, the study has provided scientific evidence for future studies, as well as for contaminant monitoring, risk management and policymaking.

## Conclusions

Our study determined that there was no increased health risk among adult residents in Shanghai, China, considering recent total cadmium exposure. Because studies have shown that primary foodstuffs, such as rice in Asian countries and tubers in Western countries, may be the main source of dietary cadmium exposure, controlling cadmium levels in these foods may be a reasonable way to control cadmium intake for non-occupational exposure. A good correlation between internal and external cadmium exposure demonstrated that combining food survey and food contamination detection could provide a reliable estimation for environmental contamination exposure. Moreover, the data in this study demonstrated that tobacco cadmium exposure was as important as dietary cadmium exposure in smokers. Therefore, increased concern regarding tobacco processing and smoking cessation could also reduce cadmium exposure. Further studies could be designed to include other susceptible populations, such as children and pregnant women, considering the additive effect of other contaminants.

## Competing interests

The authors declare that they have no competing interests.

## Authors’ contributions

PH developed the research question, collected the population-based data and wrote the paper. LYH aided in data analysis and wrote the draft manuscript. YHL and BC interviewed participants. MW performed UCd and BCd measurements. LSG, GSH and TYJ conceived and managed the project, assisted with data analysis, read all versions of the manuscript and revised them significantly. All authors read and contributed to the final version of the manuscript.

## Pre-publication history

The pre-publication history for this paper can be accessed here:

http://www.biomedcentral.com/1471-2458/13/590/prepub
